# To 3D or Not to 3D, That Is the Question: Do 3D Surface Analyses Improve the Ecomorphological Power of the Distal Femur in Placental Mammals?

**DOI:** 10.1371/journal.pone.0091719

**Published:** 2014-03-14

**Authors:** Francois D. H. Gould

**Affiliations:** Center for Functional Anatomy and Evolution, Johns Hopkins University, Baltimore, Maryland, United States of America; Monash University, Australia

## Abstract

Improvements in three-dimensional imaging technologies have renewed interest in the study of functional and ecological morphology. Quantitative approaches to shape analysis are used increasingly to study form-function relationships. These methods are computationally intensive, technically demanding, and time-consuming, which may limit sampling potential. There have been few side-by-side comparisons of the effectiveness of such approaches relative to more traditional analyses using linear measurements and ratios. Morphological variation in the distal femur of mammals has been shown to reflect differences in locomotor modes across clades. Thus I tested whether a geometric morphometric analysis of surface shape was superior to a multivariate analysis of ratios for describing ecomorphological patterns in distal femoral variation. A sample of 164 mammalian specimens from 44 genera was assembled. Each genus was assigned to one of six locomotor categories. The same hypotheses were tested using two methods. Six linear measurements of the distal femur were taken with calipers, from which four ratios were calculated. A 3D model was generated with a laser scanner, and analyzed using three dimensional geometric morphometrics. Locomotor category significantly predicted variation in distal femoral morphology in both analyses. Effect size was larger in the geometric morphometric analysis than in the analysis of ratios. Ordination reveals a similar pattern with arboreal and cursorial taxa as extremes on a continuum of morphologies in both analyses. Discriminant functions calculated from the geometric morphometric analysis were more accurate than those calculated from ratios. Both analysis of ratios and geometric morphometric surface analysis reveal similar, biologically meaningful relationships between distal femoral shape and locomotor mode. The functional signal from the morphology is slightly higher in the geometric morphometric analysis. The practical costs of conducting these sorts of analyses should be weighed against potentially slight increases in power when designing protocols for ecomorphological studies.

## Introduction

In recent years, the growing availability of computed tomography (CT) scans and surface laser scanners has reinvigorated the study of morphology [1], [2]. These tools allow high resolution three-dimensional (3D) virtual models of specimens to be collected, with which researchers have been able to develop novel ways of quantifying complex aspects of morphology, such as angles between joint surfaces [3]. The developments of the toolkit of geometric morphometrics [4] and its extension to the analysis of 3D surfaces [5], [6] have allowed the shape of complete anatomical structures to be compared directly.

However, 3D digitizing approaches come with practical costs. They are more time consuming than more traditional methods of data collection such as caliper measures, and require a considerable investment in computational hardware and techniques. CT scanners are not portable technologies, and so specimens in remote locations may be difficult to add to an analysis, making this a particular problem for studies of fossils or rare taxa. Thus, it is worth investigating whether or not the data produced by these approaches result in analyses with improved statistical performance compared to more traditional approaches.

Ecomorphology, the study of the relationship between morphology and ecology, provides an ideal arena in which to test this question. Increasing statistical power is important in ecomorphological analysis, as clear patterns of separation and prediction are vital if the morphology being studied is to be useful in understanding and reconstructing changes in the environment or ecology of taxa [7]. Thus ecomorphology has long made use of multivariate analysis of linear measurements in elucidating the relationship between form and function [8]–[10]. Studies examining 3D shape models of morphological features have so far shown promising results [11]–[13]. However, to date the two approaches have not been compared side by side, though linear measurements are still frequently used [14].

In this study I compare the effectiveness of a multivariate analysis of ratios of linear measurements with a geometric morphometric surface analysis for distinguishing locomotor modes in mammals based on distal femoral morphology. The distal femur has been used extensively in ecomorphological studies relating ungulate limb morphology to habitat type [15]–[17]. The distal femur is however a complex three-dimensional structure with smooth, regular boundaries, and as such many aspects of its shape, in particular its complex curvature, are difficult to quantify by traditional means. This is problematic as comparative anatomists have noted qualitatively that those aspects of shape information vary between mammals with different modes of locomotion [12]. Thus, the application of quantitative 3D surface analysis to the distal femur may significantly increase the amount of morphological information incorporated in the analysis. For these reasons, this is a good question for the study of the power of analyses based on 3D models of complex morphologies.

## Materials and Methods

### Ethics statement

All specimens used were part of the permanent collection of the United States Museum of Natural History, Washington DC, where they are accessible to all researchers. A complete list of all specimens used in the analysis is found in the supplemental information (Table S1).

### Samples

A sample of 44 genera of placental mammals represented by 164 individual specimens (Table 1) was assembled from the collections of the United States National Museum of Natural History (USNM). Wherever possible, multiple specimens of each genus were included in the analysis. Specimens were selected based on the availability of disarticulated distal femora which showed no signs of pathology and where the entire distal articular surface was visible. Wild caught specimens were selected over zoo specimens wherever possible. The sample covered a broad range taxonomically (five orders of placental mammals are represented) and morphologically (body size varies by several orders of magnitude). The genus was chosen as the unit of analysis as the ultimate goal of this research was to study paleontological taxa whose species level taxonomy is uncertain. Each genus was assigned to one of six locomotor modes based on the literature (Table 2).

**Table 1:** Taxa used in the analyses and their locomotor classification.

Taxon	LM	n	Behavior references
ARTIODACTYLA			
*Tragulus napu*	C	5	[44], [50]
*Pecari tajacu*	C	3	[44], [51]
*Hexaprotodon liberiensis*	T	2	[40]
CARNIVORA			
*Arctictis binturong*	A	3	[52], [53]
*Paradoxurus hermaphroditus*	A	5	[52], [53]
*Ailurus fulgens*	S	5	[52], [54]
*Nasua narica*	S	3	[50], [51]
*Bassariscus astutus*	S	5	[55]
*Potos flavus*	A	5	[50], [51]
*Procyon lotor*	S	5	[39], [53]
*Taxidea taxus*	Tf	4	[39], [51]
*Meles meles*	Tf	3	[38], [53]
*Lutra lutra*	Taq	1	[38], [40]
*Lontra canadensis*	Taq	5	[39], [40]
*Gulo gulo*	S	5	[38], [39]
*Martes pennanti*	S	5	[39], [53]
*erpestes edwardsii*	T	5	[52], [56], [57]
*Viverra zibetha*	T	2	[44], [52]
*Fossa fossana*	T	1	[44], [57]
*Eira barbara*	S	5	[50], [58]
*Vulpes zerda*	C	5	[44], [59]
*Canis latrans*	C	3	[39]
*Speothos venaticus*	T	5	[53], [60]
*Ursus americanus*	S	3	[39], [53]
*HYRACOIDEA*			
*Procavia capensis*	S	5	[44], [56]
*ERISSODACTYLA*			
*Tapirus terrestris*	T	4	[44], [51]
*ODENTIA*			
*Aplodontia rufa*	Tf	2	[39], [44]
*Ondatra rivalica*	Taq	6	[39], [40], [51]
*Petaurista petaurista*	A	4	[44], [52]
*Ratufa bicolor*	A	3	[44], [52]
*Sciurus carolinensis*	A	3	[19], [39]
*Marmota monax*	Tf	4	[19], [39]
*Castor canadensis*	Taq	4	[39], [53]
*Erethizon dorsatum*	A	4	[19], [39]
*Coendou prehensilis*	A	4	[51], [61]
*Dolichotis salinicola*	C	1	[50], [62]
*Kerodon rupestris*	S	2	[40]
*Dasyprocta azarea*	C	6	[61], [63]
*Agouti paca*	T	5	[51], [63]
*Lagostomus maximus*	Tf	4	[50], [63]
*Cavia porcellus*	T	3	[44], [50]
*Hydrochoerus hydrochoerus*	T	2	[63]
*Cynomys ludovicianus*	Tf	4	[39], [40]
*Ctenomys magellanicus*	Tf	1	[61], [63]

Legend: LM: Locomotor modes. A: arboreal; S: scansorial; T: terrestrial; Taq: semi-aquatic; Tf: semi-fossorial; C: cursorial. n: number of specimens.

**Table 2:** Locomotor categories and definitions.

locomotor mode	abbreviation	definition
Arboreal	A	Resides almost exclusively in trees
Scansorial	S	Makes use of both ground level and inclined/vertical substrates. May show substrate use specialization (e.g. ground foraging, tree nesting)
Terrestrial	T	Uses almost exclusively ground level substrate. Does not locomote fast for sustained periods of time.
Semi-aquatic	Taq	Makes use of ground and aquatic substrate. When in water is capable of underwater swimming.
Semi-fossorial	Tf	Digs burrows larger than itself.
Cursorial	C	Ground dwelling, frequently engages in sustained bouts of high speed locomotion.

### Linear measurements

**Figure 1:**
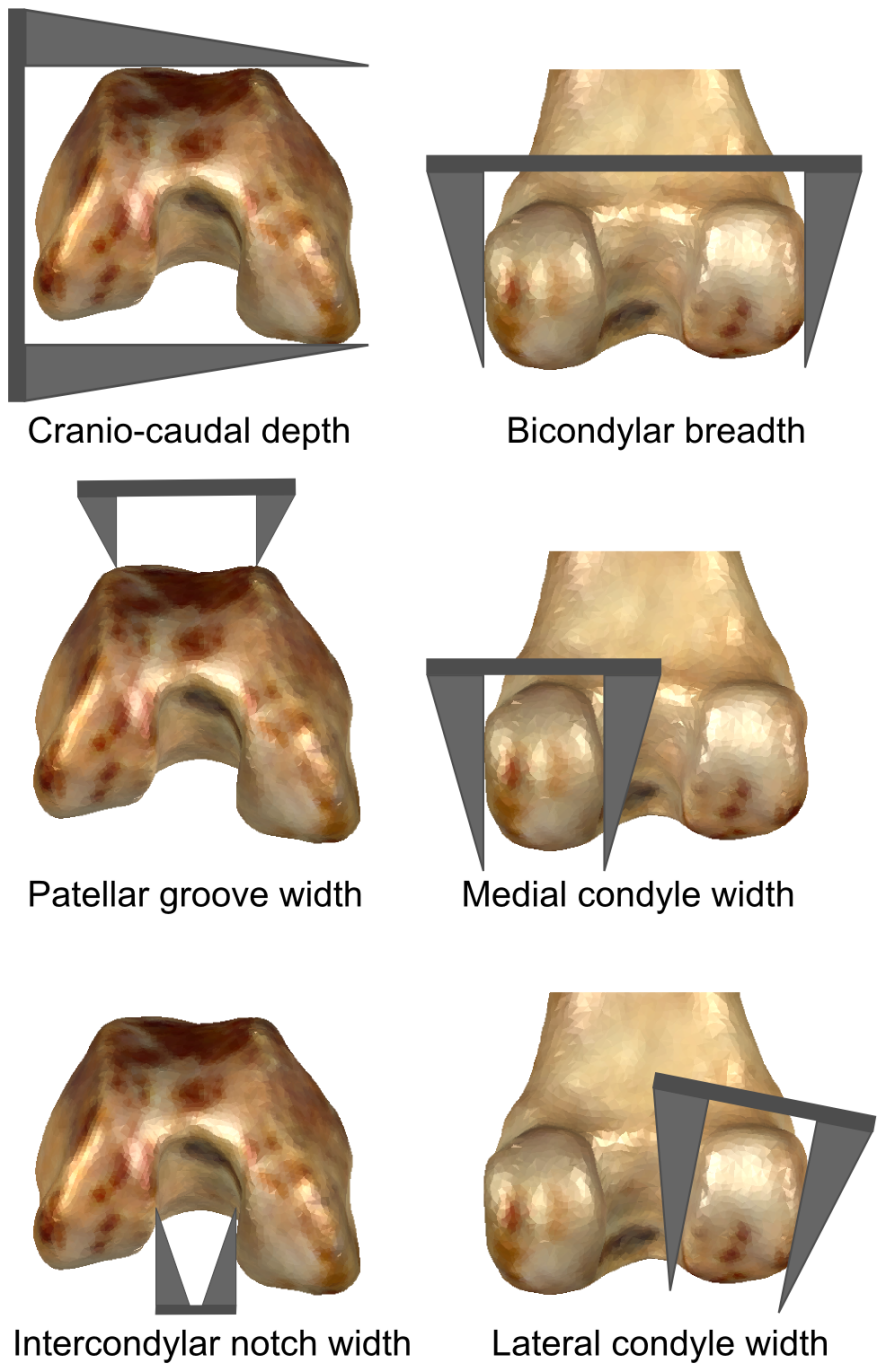
Distal femoral linear measurements. The diagrammatic representations of the calipers show their actual position when measurements were taken. The orientation of the specimens is the same as that used when taking measurements.

Six linear measurements were collected from each specimen with digital calipers (Mitutoyo corporation). Figure 1 gives detailed depictions of how each measurement was taken. The measurements were selected for their repeatability based on the results of an error study following [18] (Box S1). Thus the selected measurements are those with could be confidently repeated by the author. Measures of curvature are difficult to take from specimens, and in most studies (e.g., [19]), they are actually taken from photographs. However, the process of photography involves projecting complex three dimensional morphologies into a two dimensional plane, which can introduce difficult to quantify systematic biases [20].

Four ratios were calculated from the linear measurements (Table 3 and Table S2). The use of ratios in statistical analysis is an object of controversy (e.g., [21]). The principal problems involved with using ratios include expected skewed and leptokurtic distributions, and issues of amplified correlation between ratios and the values from which they are calculated. However, many of these issues are primarily related to the use of ratios as statistical control for confounding variables, such as body size, a function for which they are inappropriate. Conversely, they are essential for examining questions of proportionality, which are relevant to many biological problems [22]. Ratios were used in this analysis in preference to raw linear measurements for three reasons. As expressions of proportion, ratios are a better expression of shape differences described in the comparative literature [23]. Ratios are often biomechanically significant, and thus good approximations for functional properties of the feature being considered, which is particularly appropriate when studying joints. Finally, raw linear measurements in cross taxonomic studies are often highly correlated because of the large variation in body size between taxa. High autocorrelation between the variables is an undesirable property for multivariate analysis. Ratios, as expressions of proportional rather than absolute differences, are less prone to autocorrelation due to body size differences.

**Table 3:** Ratios calculated from the linear measurements.

Ratio	abbreviation
Cranio-caudal depth to bicondylar breadth	CCD/BiCondB
Patellar groove breadth to bicondylar breadth	PatGrB/BiCondB
Intercondylar notch breadth to bicondylar breadth	IntCondN/BiCondB
Medial condyle width to lateral condyle width	MedCond/LatCond

### 3D surface data collections

**Figure 2:**
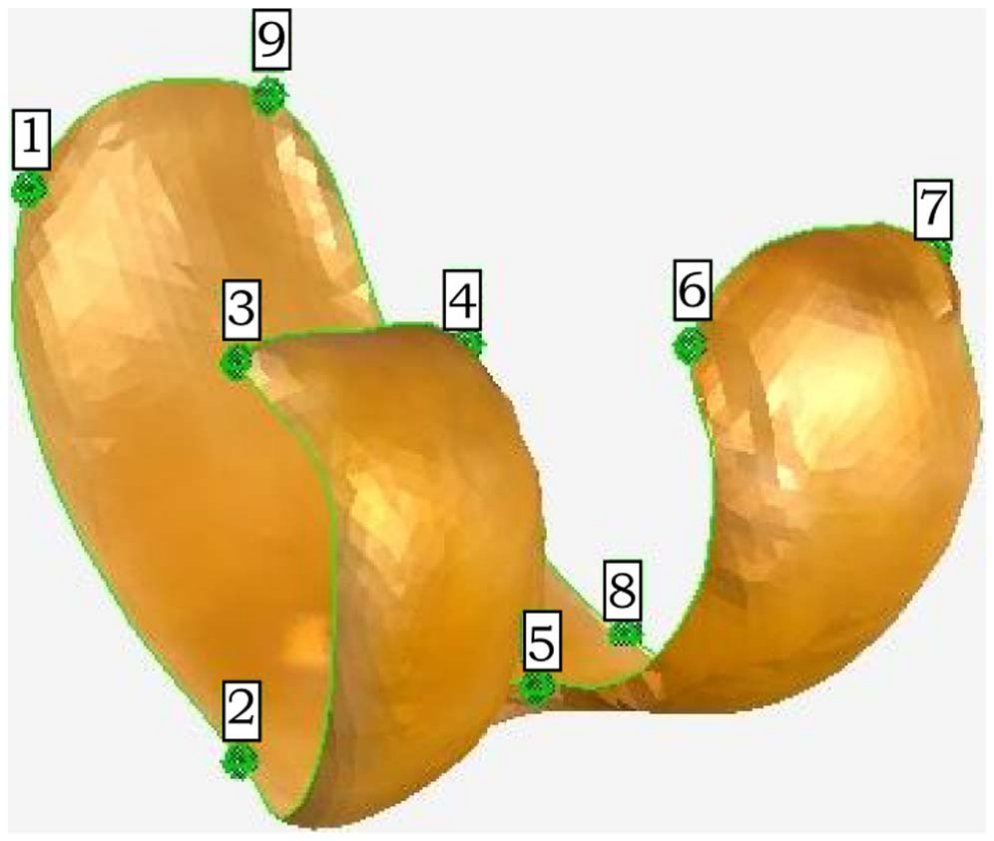
Position of landmarks placed on distal femoral surfaces models. See table 4 for details of landmark description.

All specimens were also scanned using a Next Engine portable laser surface scanner (Next Engine Corporation). The scanning protocol was derived from that used in [3]. In order to obtain all the relevant information, each specimen was scanned twice, once with the axis of rotation parallel to the shaft of the femur, and once with the axis of rotation perpendicular to the femur. Each scan family was merged into a single object in ScanStudioHD Pro (Next Engine Corporation) and exported as a. PLY file. The two objects were then imported into GeoMagic Studio 12 (Geomagic inc.), aligned and fused into a single model of the entire specimen. Any holes in the resulting mesh were filled using the GeoMagic hole-filling algorithm. The distal femoral surface was then isolated manually by delimiting a closed curve along the edge of the articular surface. Nine landmarks were sampled along the outline of each articular surface (Table 4 and Fig. 2). These are type II and type III landmarks [4], and their purpose was twofold: 1) to bring all the distal femora into a similar orientation, and 2) to break down the outline of the distal femur into broadly geometrically homologous segments to reduce the distortion of the thin plate spline in the subsequent analysis [24].

**Table 4:** Landmarks taken along the perimeter of each distal femoral surface.

	landmark	description	Landmark type
1	medial superior patellar groove	point where the superior border of the patellar groove meets the medial edge of the patellar groove	III (extremal point)
2	contact of patellar groove and medial condyle	point where the medial border of the patellar groove contacts the medial border of the medial condyle	II (maximum of curvature)
3	medial superior corner of the medial condyle	Medialmost point of the superior border of the medial condyle	III (extremal point)
4	lateral superior corner of the medial condyle	lateralmost point of the superior border of the medial condyle	III (extremal point)
5	intercondylar notch	deepest point of the intercondylar notch	II (maximum of curvature)
6	medial superior corner of the lateral condyle	medialmost point of the superior border of the lateral condyle	III (extremal point)
7	lateral superior corner of the lateral condyle	lateralmost point of the superior border of the lateral condyle	III (extremal point)
8	contact of the patellar groove and the lateral condyle	point where the lateral border of the patellar groove contacts the lateral condyle	II (maximum of curvature)
9	lateral superior patellar groove	point where the superior border of the patellar groove meets the lateral edge of the patellar groove	III (extremal point)

The isolated distal femora were imported into the matrix algebra package MatLab (MathWorks, Inc.). A MatLab algorithm that projects a standard-sized grid of 321 equally spaced points on each distal femur was written by the author. This grid size evenly samples the entire femoral surface, and provides a good representation of the femoral morphology. This grid approach allows all the specimens to be compared in a geometric morphometric analysis.

All the standardized specimens were then run through the sliding semi-landmark protocol as described in [5] through a MatLab package written by Dr. Adam Sylvester. The post-sliding coordinates of each specimen were then imported into MorphoJ [25] for geometric morphometric analysis. The specimens were brought into the same shape space through a partial Procrustes alignment. This yields Procrustes coordinates, which are the basis for geometric morphometric analysis. A variance-covariance matrix of the Procrustes coordinates was calculated, and used to calculate principal components for the original data in a manner analogous to a standard multivariate analysis. The principal components formed the input data for subsequent statistical analysis of ecomorphological hypotheses (Table S2)

### Statistics

As noted above, body size in the sample varied by several orders of magnitude. In weight bearing joints, aspects of joint shape are expected to covary allometrically with body size owing to surface to volume ratio relationships and the mechanical properties of articular cartilage [26]. Multivariate linear regression was used to assess the impact of body size on shape variation in the distal femur both in terms of statistical significance and magnitude of effect [27]. Different body size proxies were used in each analysis, in line with differences in common practice between traditional and geometric morphometrics. The ratio data were regressed against total limb length, defined as the sum of lengths of the femur, tibia, humerus and ulna [28]. The geometric morphometric data were regressed against the natural logarithm of centroid size. For the ratio data, the calculations were done in R [29]. For the geometric morphometric data, the purpose built regression function of MorphoJ was used, as it is designed to deal with large numbers of variables resulting from geometric morphometric analysis [30].

The effectiveness of each dataset at separating specimens by locomotor mode was tested using two complementary multivariate approaches. A one-way multivariate analysis of variance (MANOVA) was used to test the hypothesis that locomotor mode was correlated with distal femoral morphology. In the case of the linear measurements data, all four ratios were used. In the case of the geometric morphometric analysis, the first 24 principal components were used as they accounted for 95% of the total shape variance in the sample. Significance was estimated at α = 0.05. As well as p-values, effect size was also estimated using the η^2^ criterion in order to compare the contribution of locomotor mode to overall variance in each dataset.

Since ecomorphology is often concerned with predicting ecology from morphological variation, a discriminant function (DFA) was also used for each dataset. In each case, so as not to over-determine the model, only those univariate variables (either ratios or principal components) that were significantly correlated with locomotor mode as estimated by post-hoc ANOVAs were used to construct the DFA. The predictive power of the DFA was estimated using percent correct classification scores of the specimens. Percent correct classifications were calculated using the leave-one-out cross validation process, which removes each specimen from the training set and then classifies it as an unknown. This provides more conservative estimates of the predictive power of the DFA than simply classifying the specimens in the training set.

The significance of the percent correct classification was tested using a permutation test [31]. Permutation tests are most appropriate in this case as differences in the sizes of classes in the training set affect the null distribution of the percent correct classification. The distribution of results one expects if there is no actual difference between the members of different classes is difficult to predict when the size of classes is uneven [31]. For each dataset, specimens were randomly assigned to one of the six classes, with class sizes held constant. The discriminant function was calculated, and cross validated percent correct classification returned. This process was repeated 2,500 times to generate a null distribution of percent correct classification by locomotor mode against which the actual observed results could be compared. All statistics were calculated in R [29].

## Results

**Figure 3:**
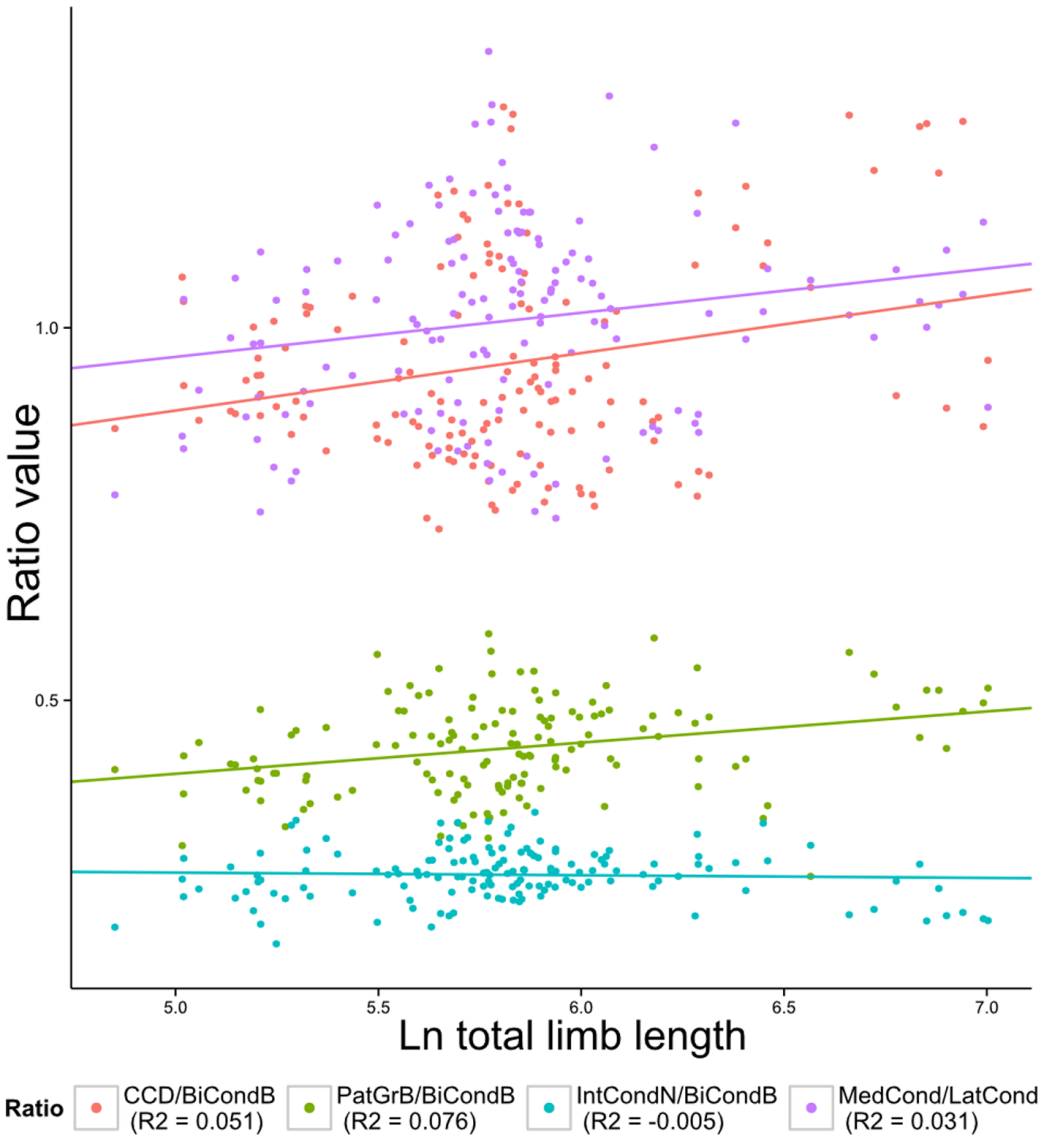
Regression of ratio variables on ln total limb length. Note that although relationships are significant, correlations (R^2^) are low.

The linear regression relationship between body size and distal femoral shape is significant for both the analysis of ratios (Table 5), and the geometric morphometric analysis (p<0.0001 based on a permutation tests with 10000 repetitions). However, correlations between ratios and total limb length are weak (Fig. 3). Similarly, the share of the variation in shape accounted for by the 24 first principal components resulting from the geometric morphometric analysis is only 5.04%. Thus, the effect of size was not considered significant enough to require formal correction.

**Table 5:** Results of the multivariate regression of ratios on ln total limb length.

Ratio	Slope	Intercept	R^2^	p-value
CCD/BiCondB	0.077	0.503	0.051	0.003
PatGrB/BiCondB	0.042	0.192	0.077	>0.001
IntCondN/BiCondB	−.0.004	0.287	−0.005	0.591
MedCond/LatCond	0.059	0.665	0.031	0.019

Locomotor mode had a very significant effect in both the analysis of ratios (Wilk's λ (4, 159) = 0.5493, p<0.001) and the analysis of principal components resulting from the geometric morphometric analysis of the entire distal femoral surface (Wilks' λ (120, 668.38)  = 0.0085, p<0.001). Effect size was larger in the geometric morphometric analysis (η^2^ = 0.52) than in the analysis of ratios (η^2^ = 0.45).

**Figure 4:**
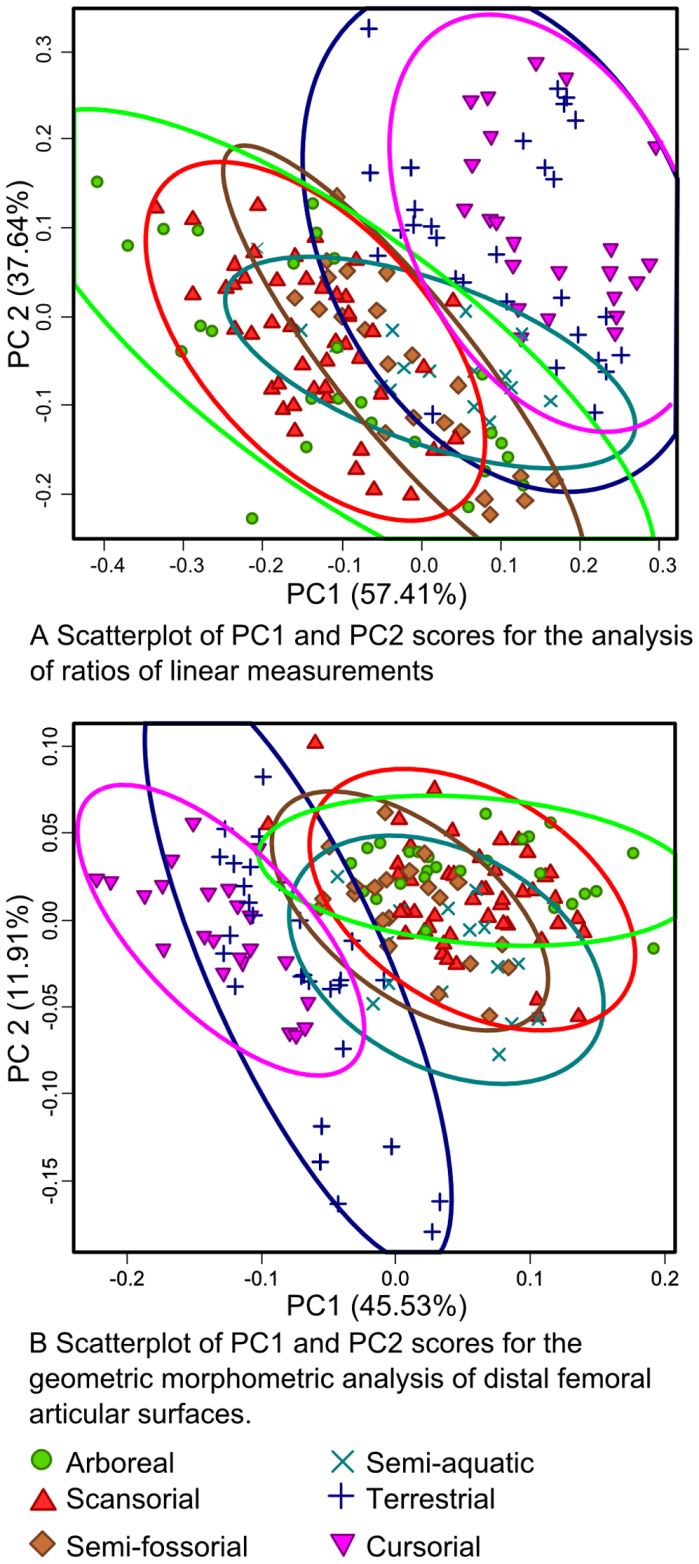
Ordination by locomotor mode of specimens for ratio and geometric morphometric analyses. Ellipses represent 95% confidence intervals on the distribution of each group. Note similar pattern in both analyses of separation of arboreal and scansorial taxa from cursorial taxa along PC1.

**Figure 5:**
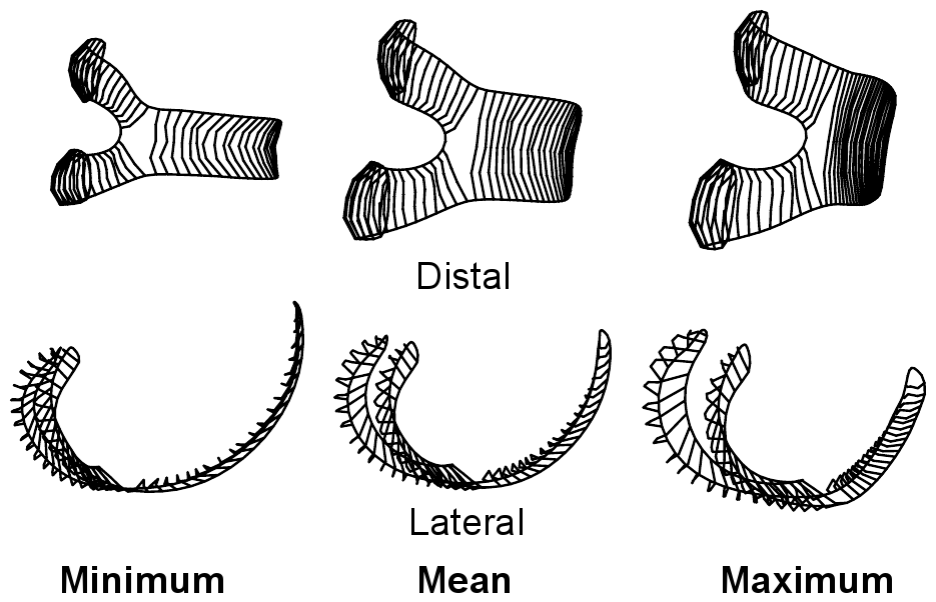
Models of correlated shape change along PC1 for the geometric morphometric analysis of distal femoral surfaces. Minimum and maximum values are minimum and maximum scores of PC1 observed in the data.

Examination of the distribution of specimens by first principal component score shows that each analysis highlights a similar pattern (Fig. 4). Morphologies are distributed along a continuum that place arboreal and cursorial taxa at opposite extremes. The 95% frequency ellipses reveal that these two groups are better separated in the geometric morphometric surface analysis than in the analysis of ratios. An examination of which ratios are correlated with principal component 1 shows a negative correlation between craniocaudal depth to bicondylar breadth and PC1 score, and a positive correlation between patellar groove breadth to bicondylar breadth and PC1 score. Modelling the aspects of correlated shape change along PC1 in the geometric analysis highlights a similar overall pattern (Fig. 5), but allows further refinements of shape difference, such as condylar shape and asymmetry and the proximal extent of the patellar groove, to be identified as significant in differentiating taxa with different locomotor modes.

**Figure 6:**
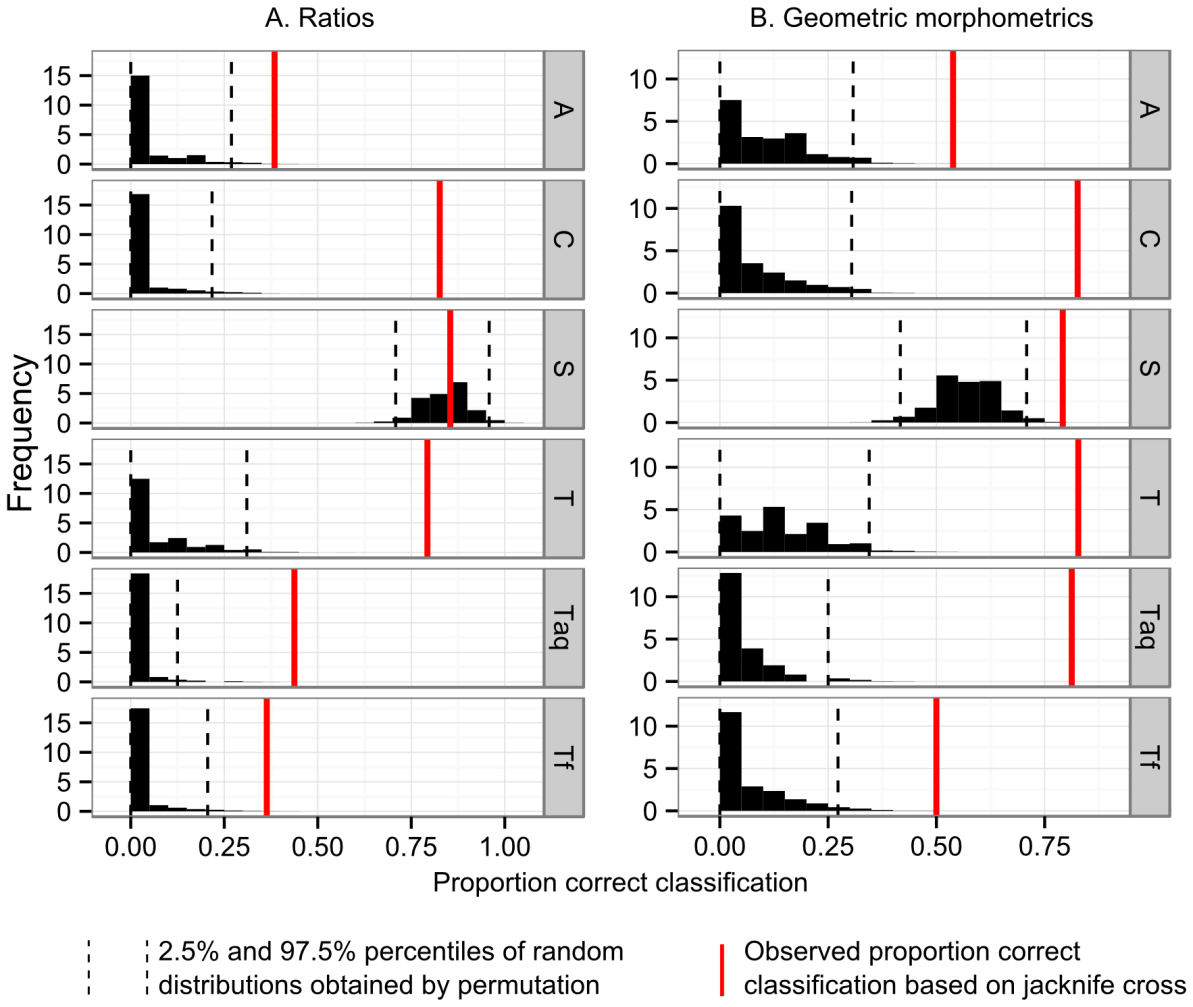
Effectiveness of observed discriminant function percent correct classification results by locomotor mode. Histograms represent distribution of percent correct classification by locomotor mode obtained from 2,500 random permutations of the data. Dashed lines are upper and lower bounds on the 95% frequency distribution. Red solid lines represent observed results. A: arboreal, C: cursorial, S: scansorial, T: terrestrial, Taq: semi-aquatic, Tf: semi-fossorial

Post-hoc univariate analyses of the variables in the MANOVA show that only three ratios are significantly correlated with locomotor mode (PatGrB/BiCondB F(1,63) = 71.979, p<0.0001; CCD/BiCondB F(1,63) = 73.351, p<0.0001; and MedCondW/LatCondW F(1,163) = 11.811, p<0.001), thus only they were used to build a discriminant function. Percent correct classification was high in some categories, low in others (Table 6). The results of the permutation test indicate that the high percent correct classification in the scansorial locomotor category is no greater than what would be expected by chance, given the differences in group size (Fig. 6A).

**Table 6:** Percent correct classification by locomotor mode obtained from leave-one-out of the discriminant functions based on the geometric morphometric analysis of surfaces and the analysis based on linear measurements.

	Percent correct classification	
Locomotor mode	Geometric morphometrics	Linear measurements
A	53.85	38.46
S	79.17	85.42
T	82.76	79.31
Taq	81.25	43.75
Tf	50.00	36.36
C	82.61	82.61

In the analysis of geometric morphometric data, 10 principal components were retained for the discriminant function analysis (PC 1, 2, 3, 4, 6, 7, 14, 16, 18, and 20, Table 7), following post-hoc univariate analysis of the variables in the MANOVA. Percent correct classification scores are higher than those obtained from the ratio based prediction (Table 6). The results of the permutation test indicate that for all categories, the predictions obtained from the geometric morphometric variables are better than chance alone would predict (Fig. 6B).

**Table 7:** Results of tests of significance for effect of locomotor mode on individual principal components from the geometric morphometric analysis of surfaces.

Principal component	F statistic	P-value
PC1	63.495	<0.0001
PC2	9.037	<0.0001
PC3	18.059	<0.0001
PC4	4.957	0.0003
PC6	4.397	0.0009
PC7	4.141	0.0015
PC14	4.288	0.0011
PC16	3.201	0.0088
PC18	4.509	0.0007
PC20	3.671	0.0036

## Discussion

### Functional significance of differences in distal femoral morphology

**Figure 7:**
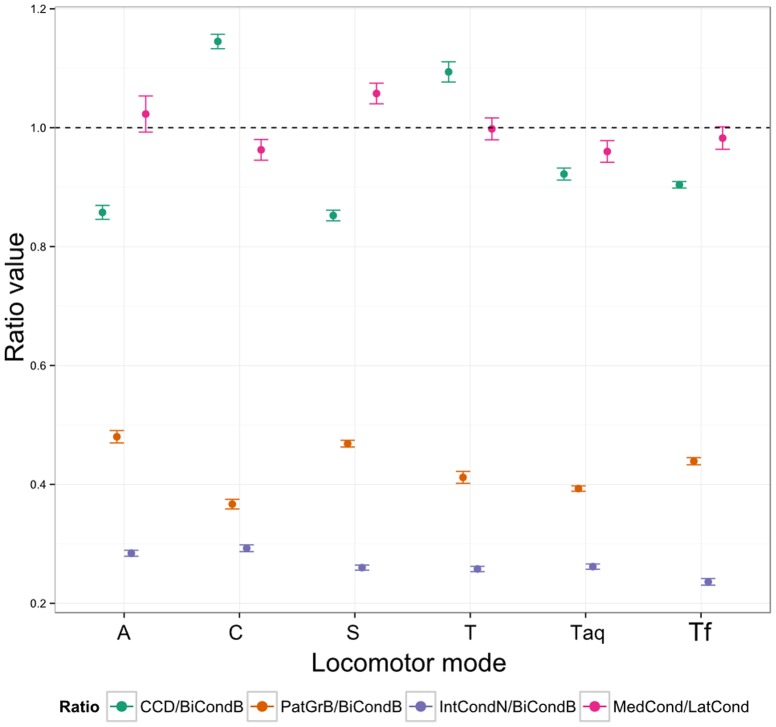
Distribution of mean and 95% confidence intervals for all for ratios by locomotor mode. Dashed horizontal line represents a ratio of 1. A: arboreal, C: cursorial, S: scansorial, T: terrestrial, Taq: semi-aquatic, Tf: semi-fossorial.

Both analyses reveal significant differences in distal femoral shape among different locomotor modes in a broad sample of extant eutherian mammals. Figure 7 summarizes the main differences between locomotor groups in the ratios. Arboreal and scansorial taxa have a ratio of craniocaudal breadth to bicondylar breadth of less than one (that is, the distal femur is mediolaterally wider than it is craniocaudally deep), whereas it is greater than 1 in cursorial and terrestrial taxa (that is, the distal femur is craniocaudally deeper than it is mediolaterally wide). The patellar groove to bicondylar breadth ration is greater in arboreal and scansorial taxa than in cursorial taxa, which indicates that the patellar groove is relatively broader in arboreal taxa, and relatively narrower in cursorial taxa. Finally, the ratio of medial to lateral condylar width is slightly greater than 1 in arboreal taxa (the medial condyle slightly wider than the lateral condyle), and slightly less than one in all other groups (the medial condyle is slightly narrower than the lateral one). This result confirms on a broader scale observations of differences in the relative widths of the condyles between arboreal and terrestrial tree shrews [32], but also reinforces that asymmetry in condylar width in eutherians is slight, unlike the situation in marsupials [33].

**Figure 8:**
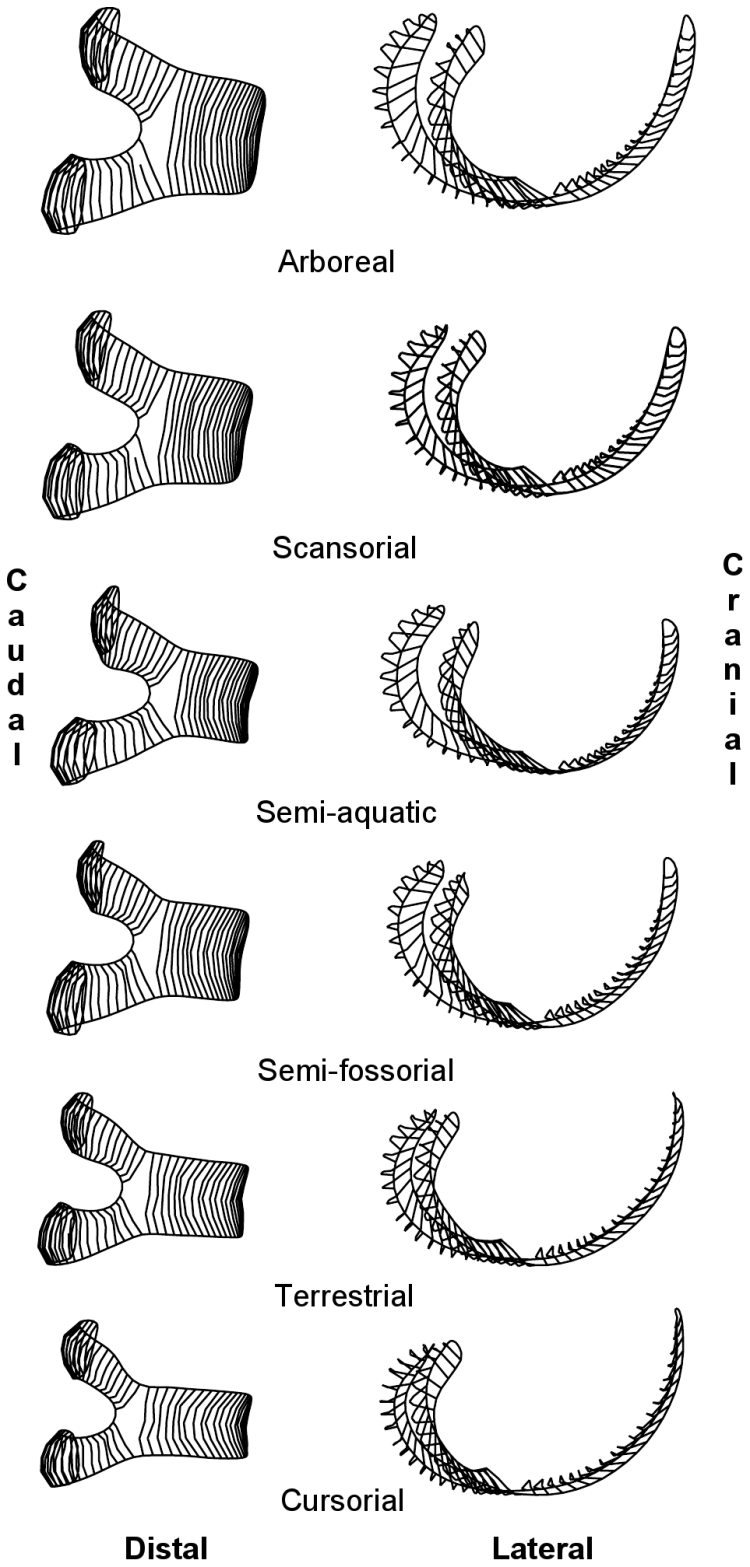
Models of group means of distal femoral morphologies for locomotor categories from the geometric morphometric analysis.

The models of mean shape by locomotor mode that were generated from the semi-landmark analysis of distal femora (Fig. 8) support the results from the ratio analysis, but are also a source of significant additional information on differences in femoral morphology among locomotor modes. Arboreal femora do indeed have broad patellar grooves relative to bicondylar breadth, a feature also found in semi-fossorial taxa, whereas cursorial taxa, and to a lesser extent terrestrial taxa, are characterized by a patellar groove that is much narrower relative to the bicondylar breadth. Interestingly, although the models do support the view that cursorial- and terrestrial-type femora are much deeper than they are wide, the clear pattern that arboreal taxa are wider than they are deep is not so apparent in the three-dimensional models. This is probably due to how antero-posterior depth was measured, as opposed to how it appears in a 2D projection of a 3D model.

The 3D models show differences among groups that the ratios used in this study do not. For example, semi-aquatic, arboreal and scansorial taxa show an asymmetry in the extent of posterior projection of the condyles. This feature is present to a lesser extent in semi-fossorial taxa, and more or less absent in terrestrial and cursorial taxa. Similarly, in cursorial taxa, the antero-posterior elongation of the distal femur is associated with a proximal extension of the patellar groove, so that its proximal border projects above the proximal extent of the condyles. The patellar groove in arboreal, scansorial and semi-aquatic taxa also appears shallower, particularly in its more proximal portion, than in cursorial and terrestrial taxa, though this effect is less than might have been expected. Finally, the overall curvature of the distal femoral surface in lateral view is different between groups: in arboreal and scansorial taxa, the curve is almost circular, while it is elliptical in cursorial and terrestrial taxa. Patellar groove medio-lateral asymmetry is not recovered as a major difference among groups.

Both analyses give a similar overall picture of variation in distal femoral morphology among locomotor groups in mammals. In addition, the results confirm at a large, quantitative scale, the qualitative and semi-quantitative observations of numerous more restricted studies (e.g. [32], [34], [35]). In contrast to [32], however, I did not find that, overall, digging behaviors are associated with a femur that is deeper cranio-caudally than in is wide medio-laterally.

The morphological differences make biomechanical sense. The craniocaudal elongation of the distal femur in cursorial taxa, for example, probably relates to increasing the moment arms of the quadriceps femoris and the gastrocnemius [33], [36] The elliptical outline of the condyles in cursorial and terrestrial taxa, versus the more circular outline in arboreal taxa, reflects both the increased arc of potential motion in the parasagittal plane in the former, but also biomechanical considerations aimed at maximizing speed over power [15]. Finally, differences in condylar symmetry (both width and posterior projection) are interpretable in terms of habitual abduction of the femur. Asymmetrical condyles are generally related to lateral displacement of the load line [33]. This condition is usually connected to femur that is held somewhat abducted from the main line of the body, which is a feature of many arboreal and digging taxa. Conversely, more terrestrial taxa hold their femora in a more erect position [37], which is reflected in the more symmetrical condyles.

### The distal femur as an ecomorphological indicator

The analysis of ratios and the analysis of sliding semi-landmarks both recover differences between locomotor modes that are consistent with previous work and make sense in a biomechanical context. Furthermore, the results of the discriminant function analysis support the conclusion that the morphology can be used to make inferences about locomotor mode and substrate use. In both analyses, the overall successful percent classification is significantly better than chance. In the analysis of ratios, only certain locomotor categories (cursorial) can be reliably inferred. In the analysis of 3D surface models, the success rate was above 80% for all categories, and was higher than what would be expected by chance alone based on the permutation test.

It is also interesting to note that misclassifications are not random. Misclassified cursorial taxa, for example, are invariably recovered as terrestrial. The analysis of ratios suggests that in terms of the proportions measured, semi-fossorial and semi-aquatic taxa are hard to distinguish, through there is also a sampling bias at work. The number of semi-aquatic taxa studied was the lowest of the sample, and most of the taxa included (otters, beavers and muskrats) are also diggers [38]–[40]. However, the analysis of surfaces did discriminate between the two groups, though misclassifications of one were recovered as the other.

The validity of the patterns uncovered by this analysis and the reliability of the resulting inferences are strongly supported by the close agreement between the two analyses. Thus, the main axis of variation in both cases maximally distinguishes arboreal taxa from cursorial taxa, and the overall pattern of distribution of the other groups is similar. Of note is the clear distinction between cursorial and terrestrial type femora on the one hand, and a looser grouping of aboreal, scansorial, semi-aquatic and semi-fossorial type femora on the other. In both analyses, the ecological factor, in this case locomotor mode, accounts for a large proportion of the total morphological variation in the sample.

The existence of such a robust pattern, as evidenced by the strong statistical significance of the MANOVA, in two very different datasets analyzing the same problem is encouraging, particularly given the broad taxonomic scope of the study. This scope was necessary as the ultimate aim was to build a ‘phylogenetically blind’ ecomorphological indicator that could be applied to the study of fossil taxa whose phylogenetic relationships are unknown. Most ecomorphological indicators have been constructed with specific clades in mind [17], [19], [34], as it is often expected that the divergent histories of different clades will obscure shared patterns. However, the conservatism of the placental knee joint [41] seems to have led to great similarities in functionally similar yet phylogenetically distant taxa. Similar correlations between form and function in distantly related taxa have been found elsewhere. Broadly unrelated actinopterygian “fish” taxa can be meaningfully compared in functional and ecological terms before and after the K/T boundary event [10]. A high-level functional similarity has been found in rodent and carnivore teeth based on an analysis of differences in dental complexity between different feeding groups [42]. Thus the distal femur appears to function as an effective ‘phylogenetically independent' indicator of locomotor mode. Further study explicitly incorporating phylogenetic information would be able to determine what proportion of the remaining variance can be attributed to shared evolutionary history.

### To 3D or not to 3D

The objective of this study was to compare the relative effectiveness of a traditional multivariate analysis based on ratios of linear measurements with a geometric morphometric analysis of the entire distal femoral surface. Much important work studying locomotor ecology using morphology has relied on either univariate [14], [43] or multivariate [19] analysis of linear measurements and ratios. However, small scale geometric morphometric studies of articular surfaces have yielded interesting results [11]–[13], [44], [45]. Furthermore, the graphical outputs of these studies can be directly related to qualitative descriptions of morphological differences, which form the basis of much of the comparative anatomical work that underpins all ecomorphological analyses. They also allow biologically interesting but difficult to measure aspects of the variation, such as changes in curvature and outline, to be included in the analysis. This study is encouraging insofar as both methods uncover significant patterns in the morphological variation of the distal femur. Furthermore, the patterns are broadly consistent between the two analyses, in that they recover a morphological continuum with arboreal taxa at one end and cursorial taxa at the other. Finally, the results are entirely consistent with existing comparative anatomical work [15], [33] and current understanding of joint biomechanics [34], [35]

Aspects of distal femoral variation not incorporated in the ratio analysis as used here, such as asymmetry in the posterior projection of the condyles, appear to enhance the distinction between arboreal and cursorial taxa in the analysis of surfaces. The three-dimensional method thus provides more information on the morphology of the distal femur. Although this does not have a major effect on the pattern in principal components space, it has a large impact on the ability to build a powerful classification function. The effect size of locomotor mode is larger in the analysis of surfaces than in the analysis of linear measurements, and the resulting discriminant function analysis far more successful at correctly classifying specimens more often than is expected by chance alone. Geometric morphometric analysis of surfaces, because it contains more shape information than an analysis based on outlines or linear measurements, is better suited to categorizing and classification [6]. In this regard, the results of the permutation test are particularly significant. The uneven group distribution introduces the same biases in the null distribution in both analyses (an increased probability of a specimen being classified as scansorial even when there is no actual difference). However, the extra shape information in the geometric morphometric analysis is sufficient to allow correct classifications of specimens more often than would be expected by chance alone. This is not the case for the analysis of ratios. The shape space also reveals itself to be a useful tool for understanding differences among groups in terms of anatomical variation. Finally, unlike in methods based on photography or linear measurement, the three-dimensional nature of the structure is fully preserved.

It is worth considering whether the comparison of the two methods used in this study is a fair one, as from a statistical viewpoint, the geometric morphometric analysis includes an order of magnitude more dependent variables than the analysis of ratios. This is problematic, as discriminant function analyses are prone to over-determination by the inclusion of extra variables [46], leading to spuriously elevated percent correct classifications. Large numbers of variables are a consequence of geometric morphometric analyses. There is as yet no standard consensus on the best way to reduce the number of variables to a statistically manageable size, hence the frequent use of permutation tests. Stepwise removal techniques exist to deal with the over-determination of discriminant function analyses, but these have been heavily criticised for their arbitrariness [46], [47]. For this study, I dealt with the dimensionality problem by identifying those principal components that were significantly associated with locomotor mode, in an attempt to include only shape information that was relevant to the classification question in the discriminant function. However, the number of variables included in the discriminant function based on the geometric morphometric data was still greater by a factor of three than that included in the discriminant function based on ratio data. To mitigate this, I used a large sample, and the more conservative leave-one-out cross validation procedure, both of which have been shown to greatly reduce the spuriously large percent correct classifications that can result from testing discriminant functions on the training set [31], [47].

A potential criticism of this comparison is that the selected ratios do not attempt to quantify many of the aspects of shape contained in the geometric morphometric analysis. To an extent, this is the result of the limitations of caliper measures: there are only so many measurements that can reliably be taken from a distal femur. But linear measures also reflect *a priori* choices by the investigator about what aspects of the shape she considers relevant to the question at hand. Geometric morphometric approaches, in contrast, make no assumptions about what aspects of the shape are considered to be most relevant to the question, rather, they will reveal those aspects of shape (e.g., [12]). This fundamental difference between the two approaches should guide investigators in their choice of method [48].

Three-dimensional surface methods are no panacea. Though they are faster and easier than in the past, they remain much more labour-intensive than taking caliper measurements. Collecting the linear measurements on each specimen took about fifteen minutes. Collecting the three-dimensional scans on each specimen took just under two hours, and the post-processing added about two more to that. Furthermore, each surface specimen in this analysis is represented mathematically by a vector of 963 elements, which makes these datasets unwieldy from a statistical perspective. Although the advances in geometric morphometrics have done much to deal with the problems of examining large multivariate datasets, there are still many unresolved statistical arguments and disagreements about how best to deal with the problems they pose.

The shape space of articular surfaces is useful as an exploratory tool: Polly *et al.* [49] were able to extract from their analysis of three-dimensional variation in the topology of calcanei a simple ratio that distinguished digitigrade from unguligrade taxa. This ratio, which was easy to calculate for a much larger sample, formed the basis of a much broader ecomorphological analysis. The close match between the results of the ratio analysis in the present study with the much more labor intensive three-dimensional analysis might suggest that the extra effort is not warranted over so broad a sample. However, investigators [6], [42] have argued that improving our ability to analyze models of biological shapes in a consistent, quantitative manner is essential for the progress of the study of morphology. It is now possible to fully quantify morphospaces and morphotypes in a manner analogous to what is done for genotypes. This study shows that these types of approaches can be applied to large datasets. Thus, they can be used profitably to address evolutionary and ecological questions. Careful protocol design, as well as a consideration of the practicalities of collecting 3D surface data, should guide researchers in their choice of approach

## Supporting Information

Table S1 **List of specimens used in the analyses.**
(XLSX)

Table S2 **All principal components scores obtained from the geometric morphometric analysis and ratios used in the analyses.**
(XLSX)

Box S1 **Results of error study on the reliability of linear measurements.**
(DOCX)
