# Supplementary material for: To 3D or Not to 3D, That Is the Question: Do 3D Surface Analyses Improve the Ecomorphological Power of the Distal Femur in Placental Mammals?
Source: PLoS One. 2014 Mar 14;9(3):e91719. doi: 10.1371/journal.pone.0091719 (PMC3954759; doi:10.1371/journal.pone.0091719)
Supplement: Box S1 — Results of error study on the reliability of linear measurements. (DOCX) [file pone.0091719.s003.docx]

**Box S1: results of error study on the reliability of linear measurements**

To gauge the reliability of the linear measurements used in this analysis, the same specimens (Table S1) were measured three times on three consecutive days by the same investigator using digital calipers. In both cases, the linear measures were subjected to a mixed-mode ANOVA design, following Bailey and Byrnes (1990). This type of analysis is the best for parsing out the relative importance of error in each stage of the data collection protocol, and allows the relative error associated with each stage of the protocol to be estimated.

Table S1: List of specimens used in error study. C: osteological collections of Johns Hopkins University, S: San Diego Museum

| Specimen number | Taxon | Locomotor mode |
| --- | --- | --- |
| C 14 | *Gulo gulo* | Terretrial |
| C 17 | *Canis lupus* | Cursorial |
| S 695 | *Castor canadensis* | Semi-aquatic |
| USNM 49642 | *Potos flavus* | Arboreal |
| USNM 56470 | *Aplodontia rufa* | Semi-fossorial |

The results of a one-way model II ANOVA show that significant measurement error is associated with only two measurements, the breadth of the patellar groove and lateral condylar width (Table S2). These measurements quantified crucial parts of the femoral morphology, and so could not be eliminated. Thus the measurements were taken for each specimen as the average of three trials to improve accuracy and consistency.

Table S2: Percent measurement error associated with each linear measurement taken directly from specimens.

| linear measurements | % ME |
| --- | --- |
| Mediolateral breadth | 0.073735 |
| Craniocaudal depth | 0.099074 |
| Intercondylar notch width | 0.472368 |
| Medial condyle width | 0.170769 |
| Lateral condyle width | **1.105875** |
| Patellar groove width | **0.797199** |
